# Pericardiotomy under clamshell approach for large posterior mediastinal sarcoma: a case report

**DOI:** 10.1186/s44215-022-00008-z

**Published:** 2022-09-27

**Authors:** Nahoko Shimizu, Yugo Tanaka, Sanae Kuroda, Takeshi Inoue, Yoshimasa Maniwa

**Affiliations:** 1 Division of Thoracic Surgery, Kobe University Graduate School of Medicine, 7-5-2 Kusunoki-cho, Chuou-ku, Kobe, Hyogo 650-0017 Japan; 2 Division of Cardiovascular Surgery, Kobe University Graduate School of Medicine, 7-5-2 Kusunoki-cho, Chuou-ku, Kobe, Hyogo 650-0017 Japan

**Keywords:** Liposarcoma, Clamshell incision, Esophageal approach

## Abstract

**Background:**

Large mediastinal tumors are sometimes difficult to completely resect because they are surrounded by vital organs. We present the case of mediastinal liposarcoma successfully resected by the clamshell approach with a posterior pericardial wall incision.

**Case presentation:**

An 81-year-old man visited the hospital because of a prolonged cough. Imaging studies revealed a large tumor of the left posterior mediastinum. The tumor surrounded the left inferior pulmonary vein and was suspected of invading the left lower lobe and esophagus. Complete resection of a large mediastinal tumor invading the left inferior pulmonary vein and esophagus was achieved by adding a posterior pericardial wall incision to the clamshell approach under cardiopulmonary bypass. Histopathological diagnosis was well-differentiated liposarcoma. Postoperative course was good, and the patient is alive without any sign of recurrence for 53 months.

**Conclusions:**

This approach could represent a novel method to safely and effectively remove large mediastinal tumors invading the lung and esophagus.

## Background

Mediastinal tumors often invade or compress neighboring organs, such as the heart, great vessels, esophagus, and trachea. Among these tumors, a huge tumor located in the posterior mediastinum is particularly difficult to resect because it is behind the heart through the midsternal approach. When the tumors were approached through a lateral thoracotomy, the periesophageal and pulmonary hilum areas may be blinded by the tumor and could lead to a high risk of unexpected neighboring organ damage during surgery. Here, we present a case of huge posterior mediastinal liposarcoma invading the esophagus and left lower lobe, which resulted in successful complete resection through the clamshell approach with a posterior pericardial wall incision under cardiopulmonary bypass (CPB).

## Case presentation

**Fig. 1:**
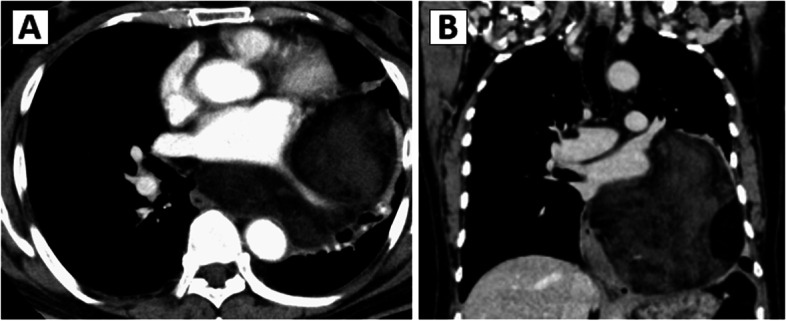
The chest computed tomography image showed a posterior mediastinal tumor measuring 160 × 140 × 120 mm displacing and invading the left lung lower lobe and esophagus

An 81-year-old man was admitted to our hospital for a prolonged cough and abnormal shadow on chest radiography. Contrast-enhanced computed tomography (CT) of the chest confirmed a large tumor (160 mm × 140 mm × 120 mm) of the left posterior mediastinum, which surrounded the left inferior pulmonary vein, and invasion of the left lower lobe and esophagus was suspected (Fig. 1). Whole body 18F-fluorodeoxyglucose (FDG) positron emission tomography/CT was performed, and there was no FDG uptake in the tumor and other organs. T2-weighted magnetic resonance imaging revealed a giant posterior mediastinal tumor with a fat component.

**Fig. 2:**
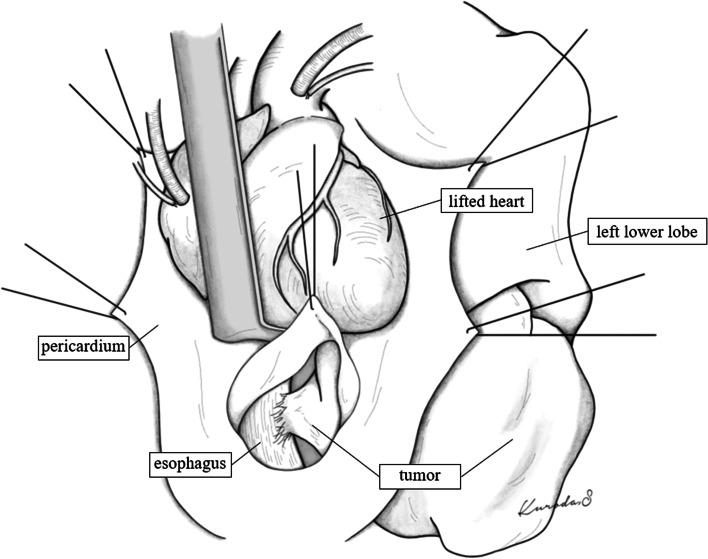
An incision of approximately 10 cm in the cranial-tail direction of the posterior pericardium revealed the positional relationship between the esophagus and tumor

The tumor was suspected to be liposarcoma, and surgery was performed with clamshell thoracotomy along the fifth intercostal space because we could recognize the relationship among the left lower pulmonary vein, esophagus, and the tumor. Intrathoracic observation revealed that the left lower lobe and heart were compressed by the tumor. Partial CPB was performed to avoid the hemodynamic instability caused by external cardiac compression to gain sufficient surgical view. The partial CPB was established with the arterial perfusion to the ascending aorta and the venous drainage from the right atrium. After initiation of CPB, the heart could be sufficiently lifted, and an incision of 10 cm in the cranial-tail direction of the posterior pericardium provided a good surgical field of view around the esophagus and allowed separation of the tumor and esophagus (Fig. 2). The tumor had infiltrated part of the esophageal wall, the outer membrane was partially resected, and the muscle layer was sutured. The tumor extended to the left lower lobe, and the inferior pulmonary vein was infiltrated by the tumor from the root. Injury to the inferior pulmonary vein during dissection caused massive bleeding. Thus, the vein was clamped and sutured intrapericardially. After resecting the tumor and left lower lobectomy, the anterior and posterior pericardium were closed with a few stitches to prevent cardiac herniation. The operative time was 512 min, and the blood loss volume was 4210 ml. The histopathological diagnosis was well-differentiated liposarcoma. The tumor invaded the inferior pulmonary vein and esophagus wall, but the surgical margin was negative. The postoperative course was good, and the patient is alive without recurrence for 53 months postoperatively.

## Discussion and conclusions

Liposarcoma arises from precursors of adipocytes, and mediastinal liposarcoma accounts for 1% of all mediastinal tumors [1]. Generally, chemotherapy and radiotherapy have limited effects on survival in sarcoma, including liposarcoma; therefore, radical surgical resection is believed to be the only curative intervention at present. However, mediastinal sarcoma is frequently massive in size and can involve or compress neighboring vital organs, and resection with enough margin is sometimes difficult. Therefore, it is necessary to examine the approach to the tumor according to tumor location and combined resecting organs.

In the present case, posterior lateral thoracotomy, hemiclamshell incision, and clamshell incision were considered as approaches. Due to the tumor volume, it was considered difficult to approach the hilar and esophagus through posterior thoracotomy without hemodynamic instability. Because the location of the tumor was predominantly in the left thoracic cavity, but it appeared to extend to the right side, a clamshell incision was selected rather than a hemiclamshell incision because of its ability to provide excellent exposure of the pulmonary hilum and mediastinum. Bains et al. [2] reported that clamshell incisions constitute an improved surgical approach for the management of the bilateral pulmonary or combined pulmonary and mediastinal disease. It is usually performed in anterior mediastinal tumor [3], but there have also been reports on a giant posterior mediastinal tumor [4].

Regarding intraoperative management, a huge mediastinal tumor sometimes obstructs the great vessels or respiratory tract and causes hypotension by external cardiac compression by the tumor and the hands of the surgeon during general anesthesia [5, 6]. In these intraoperative events, the use of cardiopulmonary assist was frequently useful in stabilizing circulatory dynamics. Conventional CPB has been established as cardiopulmonary support for such cases, but it requires 200–300 units/kg of heparin with an activated clotting time (ACT) > 400 s because it includes a venous reservoir [7]. While using partial CPB, the cardiac output is partly assisted mechanically. Therefore, the heart is beating, and some blood flow is left in the heart. It acts through the closed-loop extracorporeal circulation without blood suction or the venous reservoir, which is considered more biocompatible [8], requiring less heparin (50–100 units/kg) with an ACT of 200–250 s.

In this case, partial CPB was sufficient to stabilize intraoperative hemodynamics, and the operation could be completed safely. Moreover, in cases where the tumor has invaded the esophagus, the posterior wall of the pericardium can be fully incised by lifting the heart under the partial CPB support to obtain a good view of the esophagus and tumor. Posterior mediastinal tumor sometimes infiltrates the esophagus, trachea, and aorta, making it extremely difficult to obtain negative margins [9]. This approach allows us to perform the procedure with a sufficient field of view and contributes to complete resection in cases of suspected esophageal invasion of posterior mediastinal giant tumors.

A good visual field around the hilar structure and esophagus was obtained by adding pericardiotomy to the clamshell approach with CPB assist. This approach could be one of the useful procedures for huge posterior mediastinal tumor invading the lung and esophagus.

## Data Availability

Not applicable.

## Abbreviations

CPB: Cardiopulmonary bypass
CT: Computed tomography
FDG: Fluorodeoxyglucose
ACT: Activated clotting time
